# Design, Synthesis, and Biological Evaluation of Artemisinin-Indoloquinoline Hybrids as Potent Antiproliferative Agents

**DOI:** 10.3390/molecules191119021

**Published:** 2014-11-18

**Authors:** Li Wang, Marta Świtalska, Ning Wang, Zhen-Jun Du, Yuta Fukumoto, Nguyen Kim Diep, Ryo Kiguchi, Junzo Nokami, Joanna Wietrzyk, Tsutomu Inokuchi

**Affiliations:** 1 Division of Chemistry and Biotechnology, Graduate School of Natural Science and Technology, Okayama University, 3-1-1 Tsushima-naka, Kita-ku, Okayama 700-8530, Japan; E-Mails: liwang_512@163.com (L.W.); wn12171982@sina.com (N.W.); en422758@s.okayama-u.ac.jp (Y.F.); Nguyen-Kim.Diep@unilever.com (N.K.D.); a-k_g.dempsey.20_49@docomo.ne.jp (R.K.); 2 Institute of Immunology and Experimental Therapy, Polish Academy of Science, 12, R. Weigl Street, Wroclaw 53-114, Poland; E-Mail: switalska@iitd.pan.wroc.pl; 3 Department of Applied Chemistry, Faculty of Engineering, Okayama University of Science, Ridai-cho, Kita-ku, Okayama; 700-0005, Japan; E-Mails: duzhenjun@hotmail.com (Z.-J.D.); nogami@dac.ous.ac.jp (J.N.)

**Keywords:** artemisinin, indoloquinoline, hybrid, antiproliferative activity, cytotoxicity

## Abstract

A series of artemisinin-indoloquinoline hybrids were designed and synthesized in an attempt to develop potent and selective anti-tumor agents. Compounds **7a**–**7f**, **8** and **9** were prepared and characterized. Their antiproliferative activities against MV4-11, HCT-116, A549, and BALB/3T3 cell lines* in vitro* were tested. Nearly all of the tested compounds (**7**–**9**, except for compounds **7d** and **7e** against HCT-116) showed an increased antitumor activity against HCT-116 and A549 cell lines when compared to the dihydroartemisinin control. Especially for the artemisinin-indoloquinoline hybrid **8**, with an 11-aminopropylamino-10*H*-indolo[3,2-*b*]quinoline substituent, the antiproliferative activity against the A549 cell line had improved more than ten times. The IC_50_ value of hybrid **8** against A549 cell lines was decreased to 1.328 ± 0.586 μM, while dihydroartemisin showed IC_50_ value of >20 µM in the same cell line. Thus, these results have proven that the strategy of introducing a planar basic fused aromatic moiety, such as the indoloquinoline skeleton, could improve the antiproliferative activity and selectivity towards cancer cell lines.

## 1. Introduction

Artemisinin (**1**), a sesquiterpene lactone from *Artemisia annua*, was isolated as a result of an extensive survey for antimalarial agents in Chinese traditional herb medicines by Chinese scientists since the early 1970s [1]. Today, **1** and its derivatives, dihydroarteminisin (DHA, **2**) and artesunate (**3**), are used in the first-line treatment for multidrug-resistant malaria [2,3]. Besides the antimalarial activity, artemisinin and its semisynthetic analogs are endowed with the potential of anti-tumor [4,5,6,7], antiangiogenic [8,9], anti-inflammatory [10], anti-metastasis [11], and growth inhibition effects [12]. The most unique and important structural feature installed in artemisinin is a peroxide group in the 1,2,4-trioxane moiety, which can react with an iron complex to produce cytotoxic free radicals and selectively induces apoptosis in many high free iron level cell lines, such as cancer cells [13]. This biological sequence makes artemisinin and its analogs potent anticancer lead compounds.

**Figure 1:**
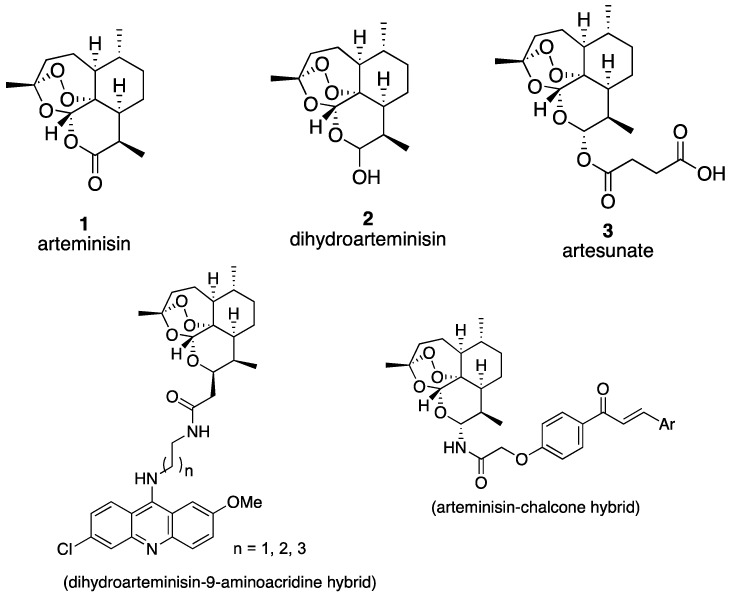
The structure of artemisinin and its analogues, and subsequent hybrid compounds.

However, compared with many traditional cancer chemotherapeutic medicines of natural origin, such as camptothecin [14], doxorubicin [15],* etc.*, simple artemisinin analogs are still less potent [4,16]. High dosage and frequent administration would be required in order to achieve the same effectiveness in the anticancer treatment due to their short half-lives. 

Many more potent artemisinin-derived antitumor agents are being developed [17,18,19]. One of the important strategies for an improved action is the generation of hybrid molecules, which involves the covalent linking of the artemisinin or its analogs with some more potent and target-selective moieties [20]. For instance, the 9-aminoacridine moiety, which is a key structure in Amsacrine, an antineoplastic agent for acute lymphoblastic leukemia, was linked with dihydroartemisinin (**2**) to form the hybrid. The antiproliferative activity of the hybrid compounds has been increased [21]. This multiple target strategy led to the design of various hybrids like artemisinin-chalcone, in which the synergistic effect of chalcone on artemisinin was demonstrated [7,22], as shown in Figure 1.

**Figure 2:**
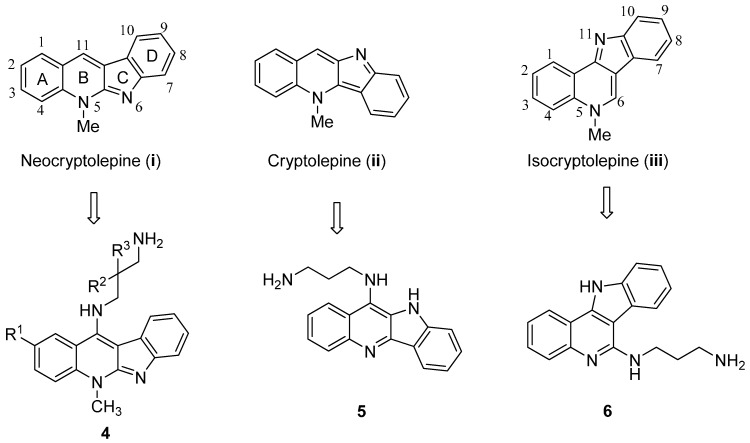
Structures of indoloquinolines from *Cryptolepis sanguinolenta* and their modifications.

The indoloquinoline skeleton has been widely found in many alkaloids [23,24,25]. In particular, alkaloids from plants are promising candidates of new lead compounds in the search for new drugs against infectious diseases and cancers [26,27]. Anticancer potential of indoloquinoline alkaloids are comprehensively documented in the recent review articles [28,29,30]. Indeed, the indoloquinoline alkaloids like neocryptolepine (**i**), cryptolepine (**ii**), and isocryptolepine (**iii**), isolated from *Cryptolepis sanguinolenta* (Lindl.) Schltr. have been used as scaffolds for drug discovery, since this plant is used as a traditional herbal medicine in West and Central Africa (Figure 2) [31,32]. Its planar fused ring system can intercalate into the DNA of tumor cells [33,34]. Besides double-helical DNA, indoloquinoline derivatives have also been found to bind DNA triplexes as well as G-quadruplexes with high affinity [35]. Thus, many indoloquinoline-featured compounds have been studied as antitumor agents [36,37,38,39,40,41,42,43]. Neocryptolepine **i** can intercalate into DNA and inhibit topoisomerase II [36]. On the other hand, cryptolepine **ii**, a regioisomer of **i**, is also a DNA intercalating agent and inhibits topoisomerase II, showing a high level of cytotoxicity [42]. A series of 5,11-dimethylindolo[2,3-b]quinolines or 6,11-dimethylindolo[2,3-b]quinolines derivatives have been tested for their chemotherapeutic activities, showing a cytotoxicity against several human cancer cell lines, with IC_50_ value ranging from 0.6 to 9 μM [44,45,46]. In these studies, the substituents, such as the Br, MeO, or Me groups, attached on the indoloquinoline core were shown to have highly infectious activities. Metal ions, such as ruthenium(II), osmium(II) or copper(II), have been introduced to the indoloquinolines scaffold for assaying their antiproliferavitve activity. Anticancer activity* in vitro* and* in vivo* have shown that the osmium(II) or copper(II) complex displayed significant growth-inhibitory activity [47,48,49]. 5H-indolo[2,3-b]quinoline (DiMIQ) derivatives containing an amino acid or a dipeptide at the C-9 position also shown impressive antitumor activity. The attachment of the hydrophilic amino acid or the peptide has increased its hydrophilic properties and made the modified DiMIQ less* in vivo* toxic and promising anticancer agent [36,43]. 

In our previous study, we also developed a series of indoloquinoline-derived antitumor agents like **4** and **6**, by modification at the C-11 position of **i** and at the C-6 position of **iii** using diverse ω-aminoalkylamino substituents (Figure 2) [43,44,45]. The target selectivity of the indoloquinoline moiety towards cancer cell lines has been well documented in these studies. Based on these facts, we are considering whether the potency of artemisinin analogues against cancer cell lines could be improved by linking to the indoloquinoline moiety. Thus, in this work, a series of dihydroartemisinin-indoloquinoline hybrids **7**–**9** were designed and synthesized. Their potencies as an antitumor agent were evaluated by antiproliferative screening.

## 2. Results and Discussion

### 2.1. Chemistry

**Scheme 1:**
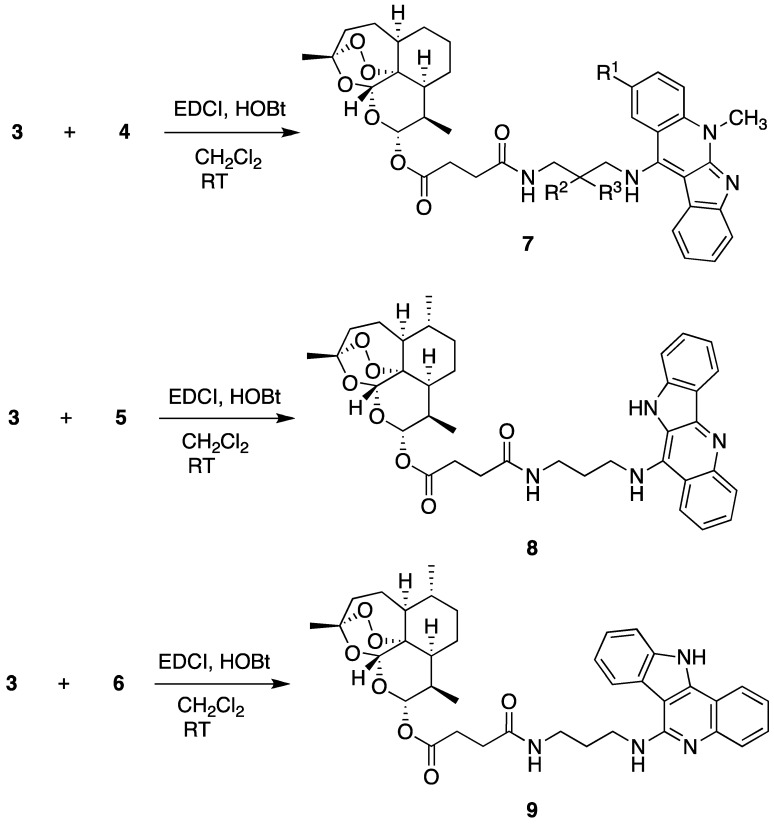
Synthetic schemes for the artesunate-indoloquinoline hybrids.

The artesunate-indoloquinoline hybrids **7**, **8** and **9** were obtained using the synthetic process shown in Scheme 1. Artesunate (**1**), which was also an artemisinin derived antimalarial agent with a better hydrophilicity, was employed as the starting material. The carboxylic acid group of the artesunate underwent condensation with ω-aminoalkylamino-indoloquinoline intermediates in the presence of 1-ethyl-3-(3-dimethylaminopropyl)carbodiimide hydrochloride (EDCl) and 1-hydroxybenzotriazole (HOBt) at room temperature to form the target compounds **7**, **8** and **9**. The preparations of the intermediate 11-(ω-aminoalkylamino)-5-methyl-5*H*-indolo[2,3-*b*]quinolines **4** and the 6-(ω-aminoalkylamino)-11*H*-indolo[3,2-*c*]quinolines **6** were carried out according to the method that we previously described in the literature [50,51,52]. The synthesis of 11-(ω-aminoalkylamino)-10*H*-indolo[3,2-*b*]quinolin **5** was carried out according to the method reported by Lavrado *et al.* [53]*.* After the appropriate purification, these hybrids were evaluated by the biological activity screening.

### 2.2. Biological Evaluation

The results of the antiproliferative activity screening for compounds **7**–**8** are summarized in Table 1 and Table 2. Biological activities of the compound **9** obtained from the isocryptolepine derivative **6** and artesunate **3** are cited in Reference [54] due to its low purity.

First, their antiproliferative activities against human leukemia cell line MV4-11 were evaluated (Table 1). All tested compounds have shown an antitumor activity with an IC50 in the microM range. Compound **7b** turned out to be the most active hybrids (IC_50_ about 0.072 μM). Compared to the corresponding amino-modified indoloquinoline intermediate **4a**, the antiproliferative activity of the artesunate-indoloquinoline hybrid **7a** was weakened, with an increased IC_50_ value from 0.066 ± 0.023 μM to 0.286 ± 0.079 μM. We also observed the same trend for the intermediate **4d** and the hybrid **7d**. Agent **9**, which is a hybrid from artesunate and 6-(3-aminopropylamino)-11*H*-indolo[3,2-*c*]quinoline (**6**), showed a better potency than agent **7a**, which reflects the difference of the counterpart in the artesunate hybrids. Compounds **7b**, **7d**, **7e**, **7f**, **8** and **9** were then selected for the next evaluation.

**Table 1:** Antiproliferative activity of the artemisinin-indoloquinoline hybrids against human leukemia MV4-11 cell line.

Compound	R_1_	R_2_	R_3_	MV4-11 ^a^ IC_50_ (μM)
cisplatin				2.820 ± 0.450
doxorubicin HCl				0.006 ± 0.002
**7a**	H	H	H	0.286 ± 0.079
**7b**	Cl	CH_3_	CH_3_	0.072 ± 0.022
**7c**	Br	CH_3_	CH_3_	0.242 ± 0.031
**7d**	CO_2_Me	H	H	0.148 ± 0.015
**7e**	CO_2_Me	CH_3_	CH_3_	0.075 ± 0.001
**7f**	CO_2_Me	H	OH	0.226 ± 0.019
**8**				0.286 ± 0.127
**4a** ^b^	H	H	H	0.066 ± 0.023
**4d** ^c^	CO_2_Me	H	H	0.086 ± 0.020
**6** ^d^				0.124 ± 0.010

^a^ MV4-11, human leukemia cell line; ^b^ Reference [50]; ^c^ Reference [51]; ^d^ Activity against MV4-11 of the compound **9** derived from **6** and artesunate **3** is cited in Reference [54].

**Table 2:** Antiproliferative activity against cancer cell lines of A549 and HCT116, and cytotoxicity against normal mice fibroblast BALB/3T3.

Compound	BALB/3T3 ^a^ IC_50_ (μM)	A549 ^b^ IC_50_ (μM)	HCT116 ^c^ IC_50_ (μM)
cisplatin	9.498 ± 0.500	8.965 ± 3.333	9.465 ± 1.300
**7b**	6.423 ± 0.996	4.555 ± 2.086	0.893 ± 0.397
**7d**	4.953 ± 0.220	3.663 ± 0.535	1.756 ± 0.329
**7e**	5.945 ± 1.163	5.060 ± 0.911	2.206 ± 0.687
**7f**	4.914 ± 0.430	4.444 ± 0.685	0.832 ± 0.216
**8**	2.725 ± 0.731	1.328 ± 0.586	0.557 ± 0.085
**4d**	0.768 ± 0.155	0.649 ± 0.080	0.130 ± 0.014
**6** ^d^	1.047 ± 0.127	0.172 ± 0.052	0.258 ± 0.107
**DHA** (**2**)	-	>20 ^e^	1.34 ± 1.06 ^e^

^a^ BALB/3T3, mouse embryonic fibroblast cell line; ^b^ A549, human non-small-cell lung adenocarcinoma cell line; ^c^ HCT116, human colon cancer cell line; ^d^ Activities against BALB/3T3, A549, and HCT116 of the compound **9** derived from **6** and artesunate **3** are cited in Reference [54]; ^e^ Reference [53].

In order to test the selectivity of these hybrids between the normal cells and cancer cells, the normal mice fibroblast BALB/3T3 cells, human lung cancer cells A549 and human colon cancer cells HCT116 were employed in the evaluation of the second step. The clinical chemotherapy drug cisplatin and antimalarial drug dihydroartemisinin were used as positive control. Generally, these tested compounds inhibited cell proliferation of these three cell lines, though the selectivity towards the HCT116 cells was better than the others. As shown in Table 2, all the tested agents (**7**–**9**) have an enhanced antiproliferative activity against the lung cancer cells A549 with IC_50_ values ranging from 1.33 to 5.06 µM, which are more active in comparison to DHA (**2**) due to the introduced planar fused ring system. Especially compound **8**, with an IC_50_ value of 1.328 ± 0.586 μM better than the both positive control, produced the best result in the screening. On the contrary, DHA (**2**) showed a poor activity against A549, with an IC_50_ of more than 20 µM. All the tested agents exhibited an antiproliferative activity superior to the positive control cisplatin. When against the HCT116 cell lines, the best result was provided by compound **8**. It inhibited the HCT116 cells proliferation with an IC_50_ of 0.557 ± 0.085 μM. 

## 3. Experimental Section 

### 3.1. General Methods

The commercially obtained reagents were directly used without further purification. The ^1^H-NMR and ^13^C-NMR spectra were measured by a Varian INOVA-600 spectrometer with CDCl_3_ as the solvent unless otherwise indicated. High-resolution mass spectra were obtained on a Bruker micrOTOF II-SKA spectrometer. The intermediate **4** and **6** were synthesized according to the method we previously reported [50,51,52]. 

#### 3.1.1. General Procedure for the Synthesis of Artesunate-Indoloquinoline Hybrids **7**

Artesunate **3** (102 mg), EDTI (39 mg) and HOBt (27.6 mg) were dissolved in CH_2_Cl_2_ with stirring for 1 h, then appropriate substituted 5-methyl-5*H*-indolo[2,3-*b*]quinoline was added with stirring together at room temperature for 6 h. TLC checked the completion of the reaction. The reaction mixture was washed by brine, dried over anhydrous MgSO_4_. After concentrated under vacuum, the crude products were purified by flash chromatography using AcOEt-MeOH (1:10 V/V) as the eluent to yield pure **7** as solids. 

#### 3.1.2. Physical Data for Compounds **7**

*Artesunate-[N^1^-(5-methyl-5H-indolo[2,3-b]quinolin-11-yl)propane-1,3-diamine] hybrid* (**7a**). Yield: 55%. ^1^H-NMR (CDCl_3_) δ 8.37 (d, *J* = 8.3 Hz, 1H), 7.91 (d, *J* = 7.7 Hz, 1H), 7.70 (d, *J* = 8.0 Hz, 1H), 7.67 (d, *J* = 7.3 Hz, 1H), 7.61 (d, *J* = 8.6 Hz, 1H), 7.37 (t, *J* = 7.6 Hz, 2H), 7.15 (t, *J* = 7.5 Hz, 1H), 6.51 (dd, *J* = 14.4, 8.0 Hz, 2H), 5.68 (d, *J* = 9.8 Hz, 1H), 5.24 (s, 1H), 4.20 (s, 3H), 3.94–3.87 (m, 1H), 3.85–3.78 (m, 1H), 3.57–3.50 (m, 1H), 3.28 (dd, *J* = 14.1, 5.7 Hz, 1H), 2.82 (dd, *J* = 9.0, 5.5 Hz, 1H), 2.74–2.69 (m, 1H), 2.55 (dd, *J* = 9.2, 5.3 Hz, 1H), 2.51–2.40 (m, 2H), 2.27 (dd, *J* = 13.9, 3.8 Hz, 1H), 1.96–1.91 (m, 1H), 1.82–1.78 (m, 1H), 1.74–1.67 (m, 2H), 1.48 (ddd, *J* = 14.7, 9.9, 3.7 Hz, 3H), 1.37–1.27 (m, 4H), 1.15 (dd, *J* = 11.4, 6.5 Hz, 1H), 1.11–0.99 (m, 2H), 0.86 (dd, *J* = 15.8, 10.0 Hz, 4H), 0.69 (d, *J* = 7.1 Hz, 3H); ^13^C-NMR (CDCl_3_) δ 172.8, 171.9, 156.9, 152.2, 148.4, 137.7, 130.4, 125.5, 124.0, 123.8, 121.9, 121.0, 118.7, 116.9, 116.5, 114.5, 106.6, 104.4, 92.3, 91.4, 80.1, 51.4, 45.0, 43.67, 37.1, 36.1, 36.0, 33.9, 32.8, 31.7, 31.3, 30.8, 29.9, 25.9, 24.4, 21.7, 20.1, 11.9. HRMS (ESI) calcd for C_38_H_47_N_4_O_7_ [M+H]^+^ Exact Mass: 671.3445, found 671.3442.

*Artesunate-[N^1^-(2-chloro-5-methyl-5H-indolo[2,3-b]quinolin-11-yl)-2,2-dimethjylpropane-1,3-diamine] hybrid* (**7b**). Yield: 47%. ^1^H NMR (CDCl_3_) δ 8.59 (d, *J* = 2.0 Hz, 1H), 7.93 (s, 1H), 7.88 (s, 1H), 7.81 (s, 1H), 7.73 (dd, *J* = 6.9, 2.3 Hz, 2H), 7.38–7.35 (m, 1H), 7.22 (ddd, *J* = 7.9, 6.8, 1.1 Hz, 1H), 7.06 (s, 1H), 5.63 (d, *J* = 9.9 Hz, 1H), 4.90 (s, 1H), 4.40 (s, 3H), 3.73–3.66 (m, 2H), 3.41–3.35 (m, 1H), 3.15–3.08 (m, 1H), 2.89 (ddd, *J* = 11.9, 7.6, 5.0 Hz, 2H), 2.72 (ddd, *J* = 13.5, 8.7, 4.8 Hz, 1H), 2.66–2.60 (m, 1H), 2.38 (ddd, *J* = 9.9, 7.1, 4.6 Hz, 1H), 2.23 (td, *J* = 14.3, 3.9 Hz, 1H), 1.92–1.85 (m, 1H), 1.74–1.67 (m, 1H), 1.39–1.33 (m, 1H), 1.31–1.27 (m, 1H), 1.23–1.13 (m, 5H), 1.05 (dd, *J* = 10.5, 4.1 Hz, 1H), 0.78 (s, 4H), 0.71 (s, 5H), 0.62 (s, 3H), 0.58 (t, *J* = 7.2 Hz, 3H); ^13^C NMR (CDCl_3_) δ 174.0, 172.2, 145.0, 143.6, 135.5, 131.7, 128.2, 126.1, 124.3, 123.5, 123.4, 122.6, 118.6, 118.1, 116.9, 114.8, 111.0, 104.2, 92.3, 91.3, 79.9, 54.3, 51.2, 46.5, 44.8, 38.8, 36.8, 36.0, 35.2, 33.8, 31.5, 30.7, 29.7, 25.7, 24.4, 24.0, 23.9, 21.5, 19.9, 11.7. HRMS (ESI) calcd for C_40_H_50_ClN_4_O_7_ [M+H]^+^ Exact Mass: 733.3368, found 733.3364.

*Artesunate-[N^1^-(2-bromo-5-methyl-5H-indolo[2,3-b]quinolin-11-yl)-2,2-dimethylpropane-1,3-diamine] hybrid* (**7c**). Yield: 43%. ^1^H-NMR (CDCl_3_) δ 8.62 (d, *J* = 7.9 Hz, 1H), 7.90 (d, *J* = 7.9 Hz, 1H), 7.79–7.72 (m, 3H), 7.55 (t, *J* = 7.6 Hz, 1H), 7.32 (t, *J* = 7.6 Hz, 1H), 7.25 (t, *J* = 7.6 Hz, 1H), 7.21 (m, 1H), 5.63 (d, *J* = 9.9 Hz, 1H), 4.92 (s, 1H), 4.38 (s, 3H), 3.73–3.67 (m, 2H), 3.32 (dd, *J* = 13.8, 6.8 Hz, 1H), 3.11 (dd, *J* = 13.9, 5.7 Hz,1H), 2.87–2.77 (m, 3H), 2.73–2.68 (m, 1H), 2.38–1.33 (m, 1H), 2.20 (td, *J* = 14.0, 3.7 Hz, 1H), 1.86–1.83 (m, 1H), 1.71–1.68 (m, 1H), 1.34 (dt, *J* = 13.1, 4.3 Hz, 1H), 1.27 (d, *J* = 11.2 Hz,1H), 1.23–1.13 (m, 5H), 1.04 (m, 1H), 0.74–0.65 (m, 6H), 0.58–0.56 (m, 6H); ^13^C NMR (CDCl_3_) δ 174.2, 172.1, 151.9, 143.6, 136.8, 132.1, 128.2, 125.8, 124.0, 123.5, 121.6, 121.3, 118.4, 117.2, 115.4, 113.9, 111.2, 104.2, 92.1, 91.2, 79.9, 60.4, 54.4, 51.2, 46.3, 44.8, 38.9, 36.8, 36.1, 35.3, 33.8, 31.5, 30.4, 29.6, 25.7, 24.4, 24.0, 23.9, 19.9, 11.7.; HRMS (ESI) calcd for C_40_H_50_BrN_4_O_7_ [M+H]^+^ Exact Mass: 777.2863, found 777.2858.

*Artesunate-[methyl 11-(3-aminopropylamino)-5-methyl-5H-indolo[2,3-b]quinoline-2-carboxylate] hybride* (**7d**). Yield: 46%. ^1^H-NMR (CDCl_3_) δ 9.09 (d, *J* = 1.7 Hz, 1H), 8.30 (dd, *J* = 8.9, 1.8 Hz, 1H), 8.01 (d, *J* = 7.7 Hz, 1H), 7.73 (d, *J* = 7.9 Hz, 1H), 7.65 (d, *J* = 9.0 Hz, 1H), 7.39 (t, *J* = 7.5 Hz, 1H), 7.21 (t, *J* = 7.2 Hz, 1H), 6.66 (s, 1H), 6.35 (s, 1H), 5.70 (d, *J* = 9.9 Hz, 1H), 5.27 (s, 1H), 4.26 (s, 3H), 4.00 (d, *J* = 3.2 Hz, 3H), 3.94 (d, *J* = 6.5 Hz, 1H), 3.88 (d, *J* = 7.0 Hz, 1H), 3.61 (d, *J* = 7.2 Hz, 1H), 3.41 (dd, *J* = 14.2, 5.8 Hz, 1H), 2.90–2.84 (m, 1H), 2.79–2.73 (m, 1H), 2.58 (dd, *J* = 8.9, 5.4 Hz, 1H), 2.54–2.50 (m, 1H), 2.50–2.45 (m, 1H), 2.35–2.28 (m, 1H ), 1.99–1.94 (m, 1H), 1.90 (dd, *J* = 10.8, 5.7 Hz, 2H), 1.82 (s, 1H), 1.61–1.55 (m, 2H), 1.52 (dd, *J* = 13.6, 4.4 Hz, 1H), 1.35 (m, 4H), 1.22–1.11 (m, 3H), 0.91 (m 4H), 0.73 (d, *J* = 7.1 Hz, 3H); ^13^C NMR (CDCl_3_) δ 172.8, 171.97, 166.7, 166.3, 140.6, 130.8, 127.4, 126.0(2C), 123.7, 122.5, 121.7(2C), 119.9, 117.3, 116.7, 115.8, 114.7, 104.5, 92.4, 91.5, 80.2, 52.6, 51.5, 45.2, 44.6, 37.3, 36.3, 36.2, 34.1, 33.4, 31.8, 31.6, 31.1, 30.1, 26.0, 24.6, 21.9, 20.3, 12.0. HRMS (ESI) calcd for C_40_H_49_N_4_O_9_ [M+H]^+^ Exact Mass: 729.3500, found 729.3502.

*Artesunate-[methyl 11-(3-amino-2,2-dimethylpropylamino)-5-methyl-5H-indolo[2,3-b]quinoline-2-carboxylate] hybrid* (**7e**). Yield: 51%. ^1^H-NMR (CDCl_3_) δ 9.27 (d, *J* = 1.7 Hz, 1H), 8.32–8.28 (m, 1H), 8.02 (d, *J* = 7.7 Hz, 1H), 7.74 (d, *J* = 7.9 Hz, 1H), 7.68–7.63 (m, 1H), 7.43–7.39 (m, 1H), 7.24–7.21 (m, 1H), 7.15 (s, 1H), 6.31–6.26 (m, 1H), 5.65 (d, *J* = 9.9 Hz, 1H), 5.09 (s, 1H), 4.26 (d, *J* = 5.1 Hz, 3H), 3.99 (s, 3H), 3.74 (ddd, *J* = 21.1, 13.5, 6.1 Hz, 2H), 3.60 (dd, *J* = 14.4, 8.0 Hz, 1H), 3.03 (dd, *J* = 14.4, 5.7 Hz, 1H), 2.97 (ddd, *J* = 17.7, 9.7, 4.8 Hz, 1H), 2.79 (ddd, *J* = 17.7, 6.2, 4.7 Hz, 1H), 2.67 (ddd, *J* = 14.5, 9.7, 4.6 Hz, 1H), 2.61–2.55 (m, 1H), 2.39 (ddd, *J* = 9.9, 7.1, 4.6 Hz, 1H), 2.28 (td, *J* = 14.3, 3.9 Hz, 1H), 1.97–1.91 (m, 1H), 1.81–1.76 (m, 1H), 1.39–1.32 (m, 3H), 1.30 (s, 3H), 1.22–1.18 (m, 1H), 1.10 (td, *J* = 11.3, 6.7 Hz, 1H), 0.93–0.88 (m, 1H), 0.86 (d, *J* = 6.2 Hz, 3H), 0.82 (s, 3H), 0.74 (t, *J* = 10.9 Hz, 2H), 0.66 (s, 3H), 0.58 (d, *J* = 7.1 Hz, 3H); ^13^C-NMR (CDCl_3_) δ 173.2, 172.1, 166.5, 164.8, 148.5, 140.4, 131.8, 130.5, 126.6, 125.3, 124.1, 122.4(2C) 122.3, 119.3, 117.2, 116.1, 114.3, 104.2, 92.3, 91.3, 80.0, 60.3, 54.2, 52.3, 51.3, 46.6, 44.8, 38.6, 36.8, 36.1, 33.8, 31.5, 30.9, 29.7, 25.8, 24.4, 24.0, 23.7, 21.4, 20.0, 11.6. HRMS (ESI) calcd for C_42_H_53_N_4_O_9_ [M+H]^+^ Exact Mass: 757.3813, found 757.3809. 

#### 3.1.3. Procedure for the Synthesis of Artesunate-Indoloquinoline Hybrid **8**

Artesunate **3** (102 mg), EDTI (39 mg) and HOBt (27.6 mg) were dissolved in CH_2_Cl_2_ with stirring for 1 h, then N^1^-(10*H*-indolo[3,2-*b*]quinolin-11-yl)propane-1,3-diamine **5** was added to the mixture with stirring together at room temperature for 6 h. TLC checked the completion of the reaction. The reaction mixture was washed by brine, dried over anhydrous MgSO_4_. After concentrated under vacuum, the crude products were purified by flash chromatography using AcOEt-MeOH (1:10 V/V) as the eluent to yield pure **8** as solid.

#### 3.1.4. Physical Data for Compound **8**

*Artesunate-[11-(3-aminopropylamino)-10H-indolo[3,2-b]quinoline] hybrid* (**8**). Yield: 40%. ^1^H-NMR (CDCl_3_) δ11.18 (br. s., 1 H), 8.04 (d, *J* = 7.63 Hz, 1 H), 8.00 (d, *J* = 8.51 Hz, 1 H), 7.91 (d, *J* = 7.92 Hz, 1 H), 7.87 (d, *J* = 8.22 Hz, 1 H), 7.78 (d, *J* = 7.92 Hz, 1 H), 7.30 - 7.39 (m, 2 H), 7.24 (m, 1 H), 7.13 (d, *J* = 7.63 Hz, 1 H), 7.07 (m, 1 H), 6.87 (t, *J* = 6.75 Hz, 1 H), 6.56 (t, *J* = 6.75 Hz, 1 H), 5.65 (d, *J* = 9.98 Hz, 1 H), 5.15 (s, 1 H), 4.02 (br. s., 1 H), 3.38–3.33 (m, 2 H), 2.78–2.69 (m, 2 H), 2.66–2.57 (m, 2 H), 2.41–2.50 (m, 1 H), 2.28–2.25 (m, 1 H), 2.01–1.97 (m, 4 H), 1.87 (d, *J* = 14.09 Hz, 1 H), 1.77–1.75 (m, 1 H), 1.56–1.51 (m, 2 H), 1.47 (dt, *J* = 13.50, 4.25 Hz, 1 H), 1.32–1.25 (m, 4 H), 1.18–1.10 (m, 1 H), 1.10–1.01 (m, 2 H), 0.92–0.80 (m, 4 H), 0.77–0.67 (d, *J* = 7.04 Hz, 3 H); ^13^C-NMR (CDCl_3_) δ 172.7, 172.0, 143.7, 142.6, 142.1, 136.3, 134.1, 129.8, 128.2, 124.4, 123.3, 121.8, 119.9, 116.1, 114.1 114.0, 112.4, 104.3, 92.2, 91.3, 80.0, 60.4, 51.3, 45.0, 37.1, 36.1, 33.9, 31.7, 30.6, 29.7, 25.8, 24.4, 21.8, 21.0, 20.1, 14.2, 12.0. HRMS (ESI) calcd for C_37_H_45_N_4_O_7_ [M+H]^+^. Exact Mass: 657.3288, found 657.3289.

### 3.2. Cell Line

MV4-11 cells were cultured in the RPMI 1640 medium (IIET, Wroclaw) supplemented with 2 mM L-glutamine and 1.0 mM sodium pyruvate, 10% fetal bovine serum (all from Sigma Aldrich, Germany). HCT116 and A549 cells were cultured in the RPMI1640 + OptiMEM (50:50) medium (IIET, Wroclaw) supplemented with 2 mM L-glutamine and 5% fetal bovine serum (all from Sigma Aldrich, Germany), BALB/3T3 cells were cultured in Dulbecco medium (IIET, Wroclaw) supplemented with 2 mM L-glutamine and 1.0 mM sodium pyruvate, 10% fetal bovine serum (all from Sigma Aldrich, Germany). All culture medium was supplemented with 100 units/mL penicillin and 100 mg/mL streptomycin (both from Polfa, Tarchomin S.A., Warsaw, Poland). All cell lines were grown at 37 °C with 5% CO_2_ humidified atmosphere.

### 3.3. Antiproliferative Assay in Vitro

Test solutions of the compounds tested (1 mg/mL) were prepared by dissolving the substances in 100 μL of DMSO completed with 900 μL of tissue culture medium. Afterward, the tested compounds were diluted in culture medium to reach the final concentrations of 10, 1, 0.1, 0.01 μg/mL. Twenty four hours prior to the addition of the tested compounds, the cells were plated in 96-well plates (Sarstedt, Germany) at a density of 1 × 10^4^ or 0.5× 10^4^ (HCT116) cells per well. The assay was performed after 72 h of exposure to varying concentrations of the tested agents. The* in vitro* cytotoxic effect of all agents was examined using the MTT (for MV4-11 cell line) or SRB (for A549, HCT116 and BALB/3T3 cell lines) assay [55]. The results were calculated as an IC_50_ (inhibitory concentration 50)–the dose of tested agent that inhibits proliferation of 50% of the cell population. IC_50_ values were calculated for each experiment separately and mean values with SD are presented in Table 1 and Table 2. Each compound in each concentration was tested in triplicate in a single experiment, which was repeated 3–5 times.

## 4. Conclusions 

In this study, we have synthesized and characterized a series of new artesunate-indoloquinoline hybrids, including compounds **7**, **8** and **9**. Their anticancer activities were evaluated by antiproliferative screening* in vitro* against the MV4-11, HCT-116, A549, and BALB/3T3 cell lines. Results have shown that nearly all of the tested compounds displayed an antiproliferative activity compared to the dihydroartemisinin. It was proved that the introduction of the indoloquinoline skeleton improved the antiproliferative activity and selectivity towards cancer cell lines of artemisinin. Further research on the modifications of indoloquinoline and use of different artemisinin analogues is still ongoing. 

## Supplementary Materials

Supplementary materials can be accessed at: http://www.mdpi.com/1420-3049/19/11/19021/s1.

## Supplementary Files

Supplementary File 1
