# Supplementary material for: Design, Synthesis, and Biological Evaluation of Artemisinin-Indoloquinoline Hybrids as Potent Antiproliferative Agents
Source: Molecules. 2014 Nov 18;19(11):19021–35. doi: 10.3390/molecules191119021 (PMC6271626; doi:10.3390/molecules191119021)

Supplementary

Figure S1. ^{1}H-NMR spectra of 7a.

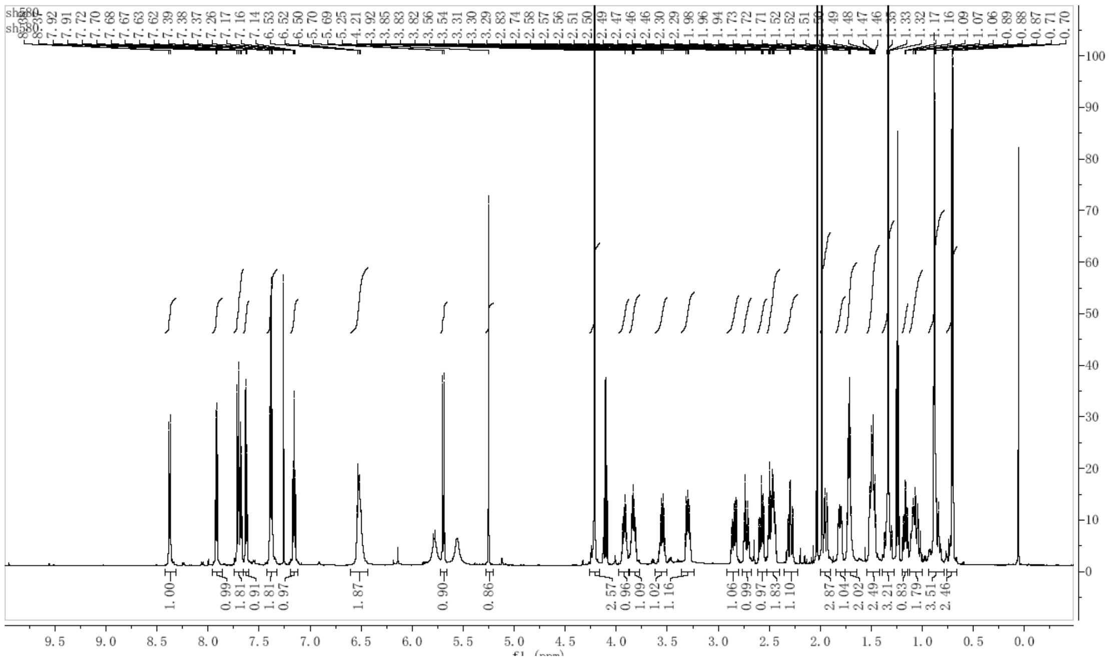

Figure S2. ^{13}C-NMR spectra of 7a.

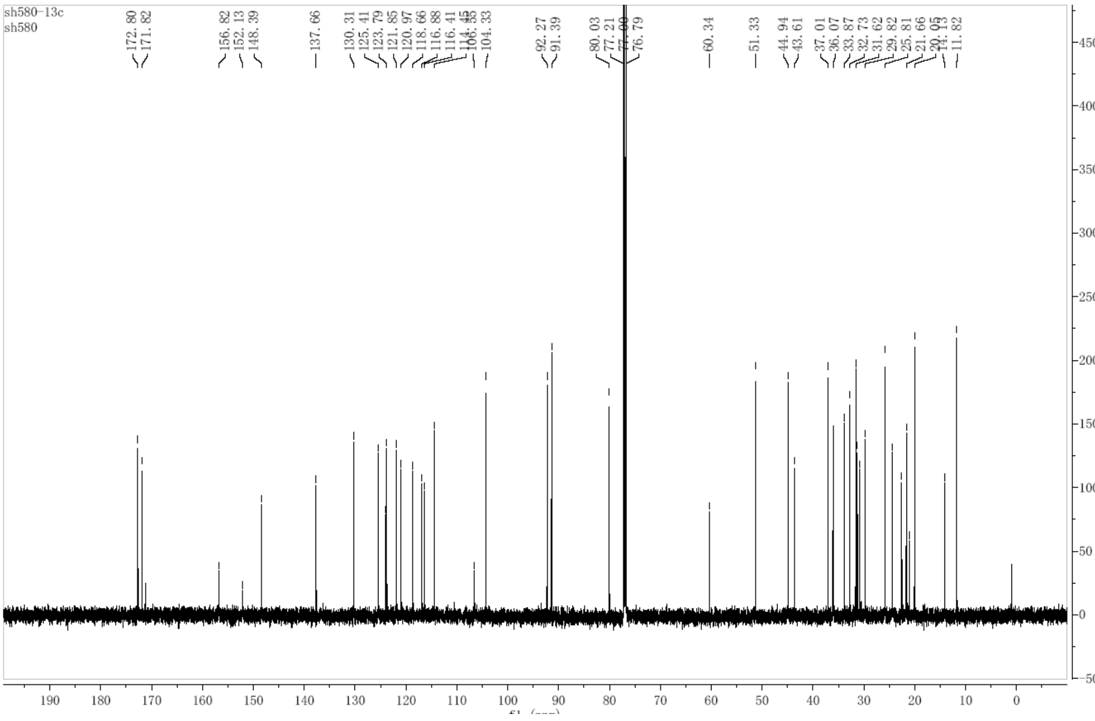

Figure S3. HRMS of 7a.

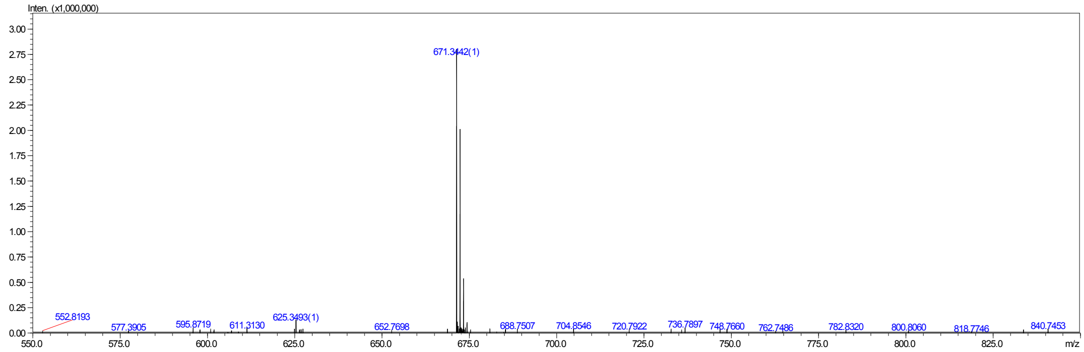

Figure S4. ^{1}H-NMR spectra of 7b.

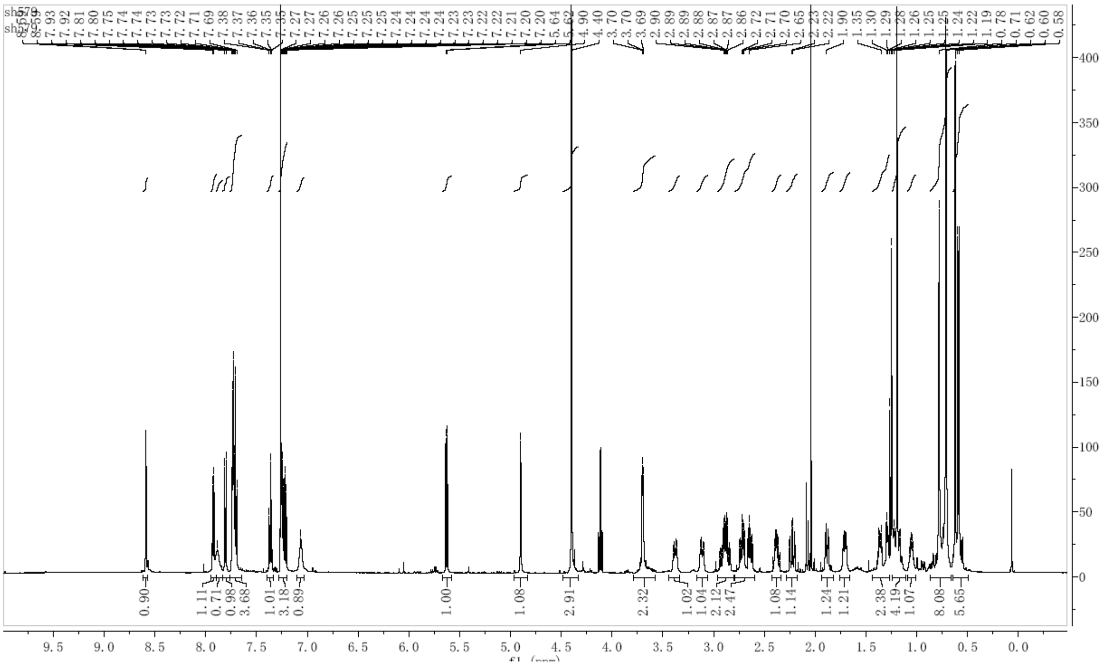

Figure S5. ^{13}C-NMR spectra of 7b.

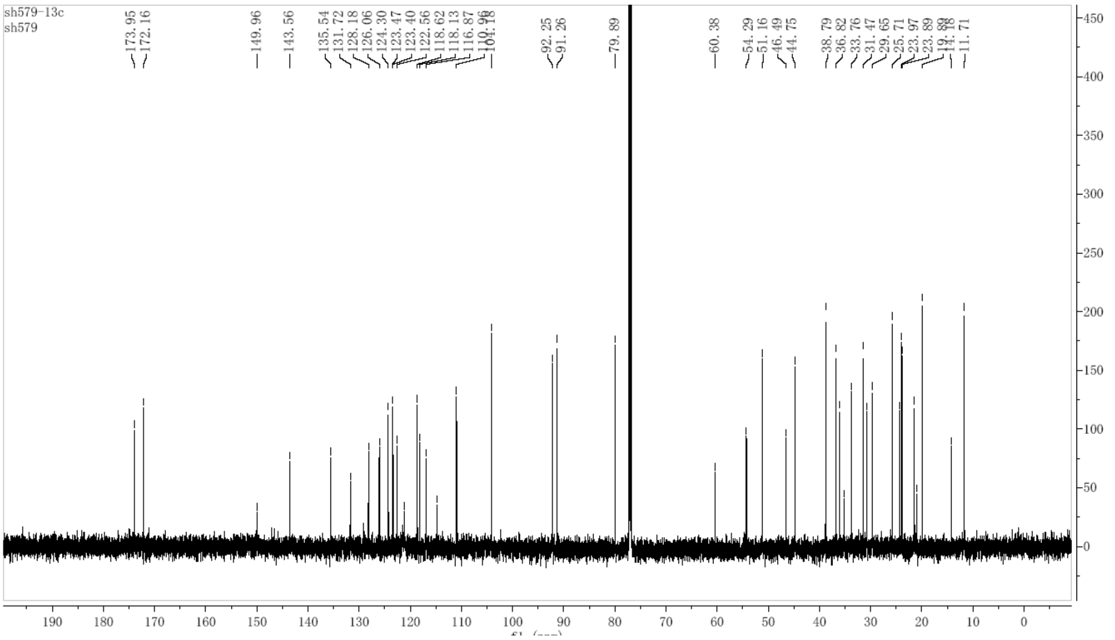

Figure S6. HRMS of 7b.

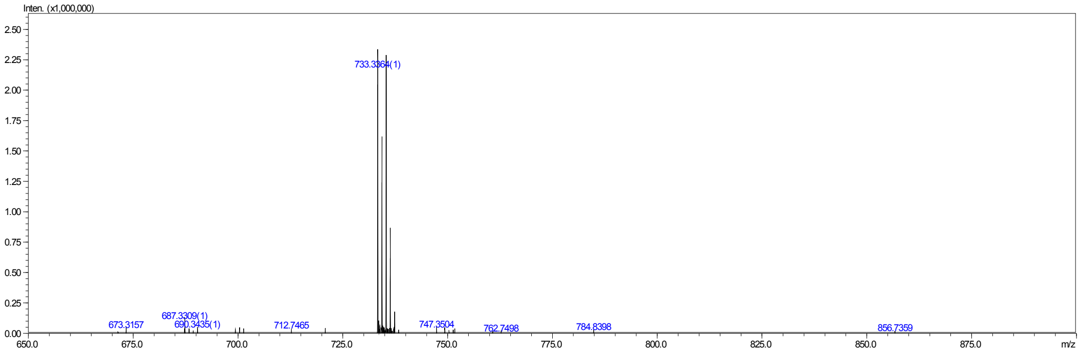

Figure S7. ^{1}H-NMR spectra of 7c.

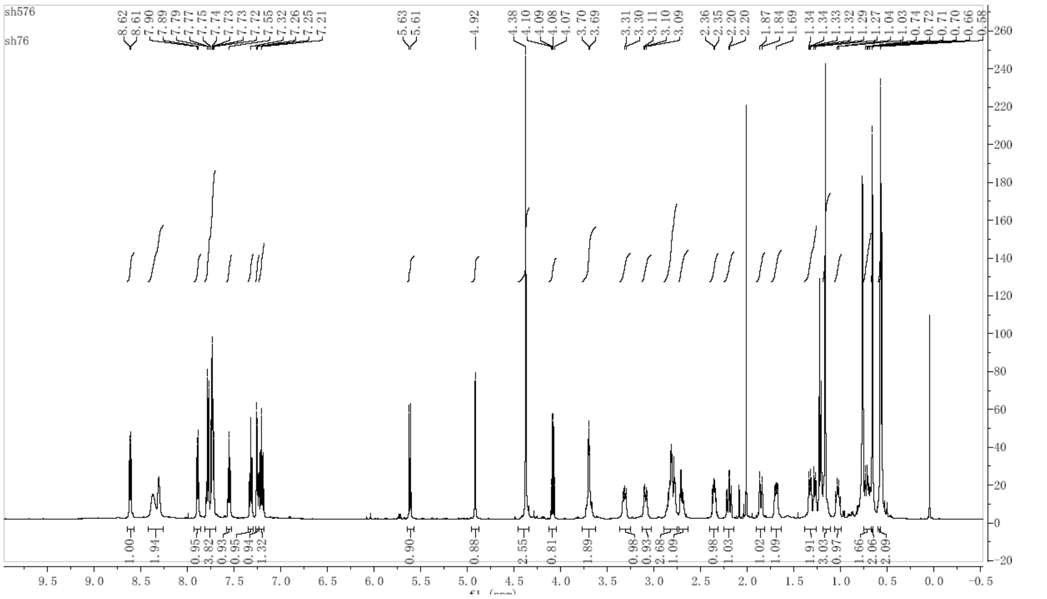

Figure S8. ^{13}C-NMR spectra of 7c.

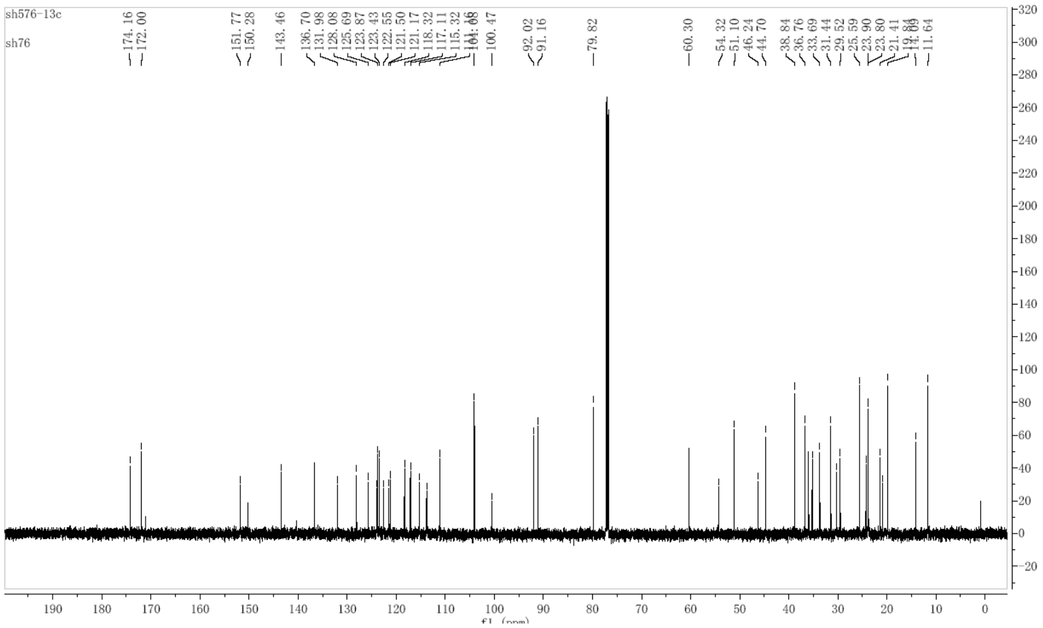

Figure S9. HRMS of 7c.

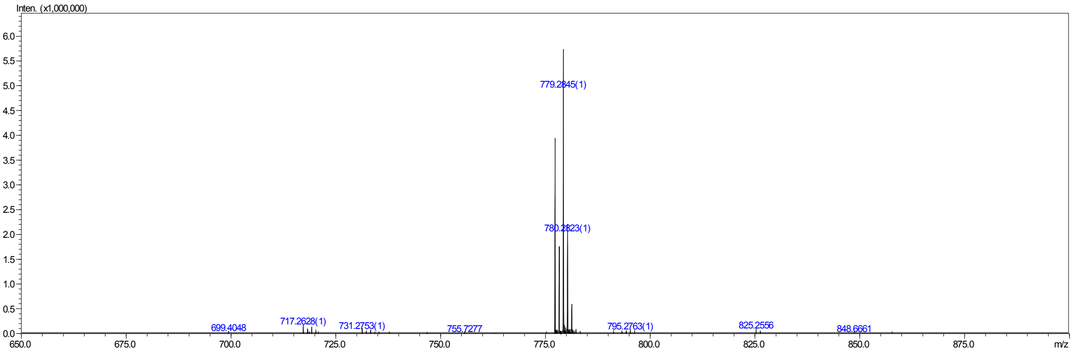

Figure S10. ^{1}H-NMR spectra of 7d.

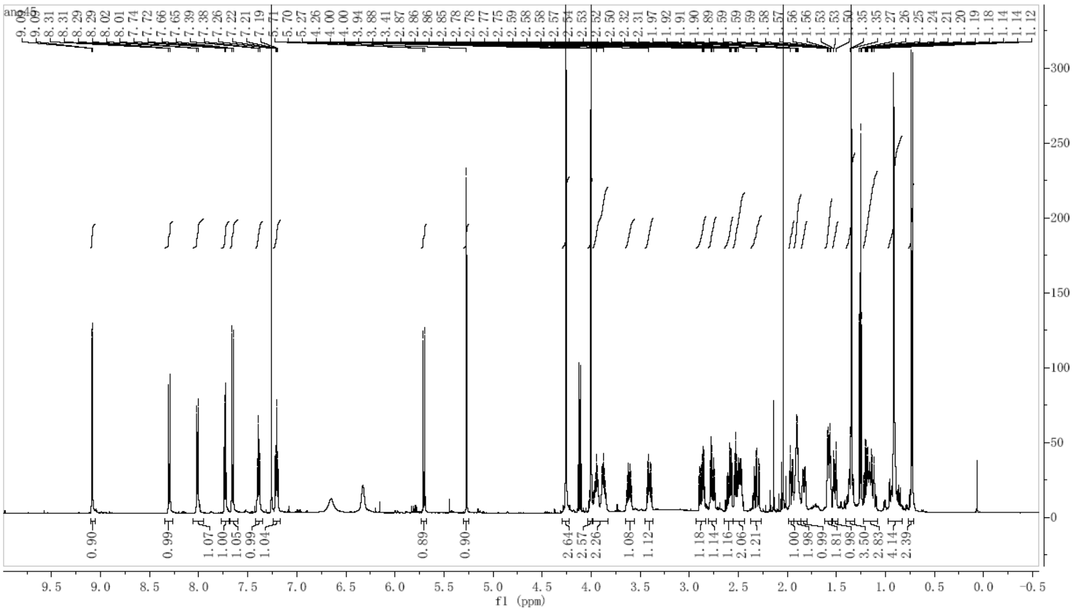

Figure S11. ^{13}C-NMR spectra of 7d.

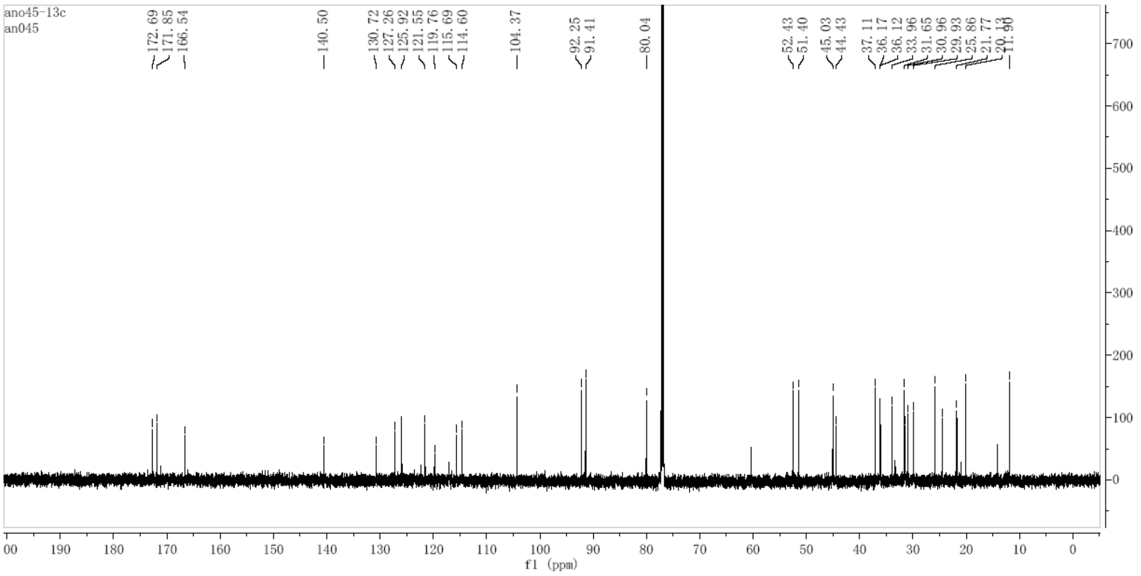

Figure S12. HRMS of 7c.

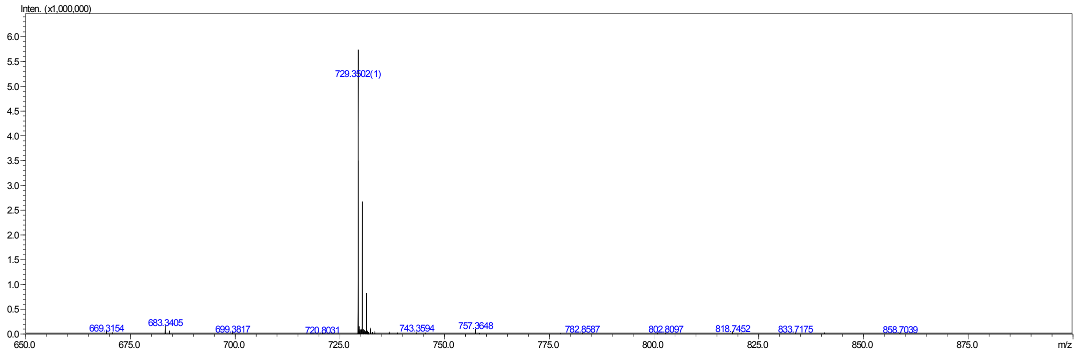

Figure S13. ^{1}H-NMR spectra of 7e.

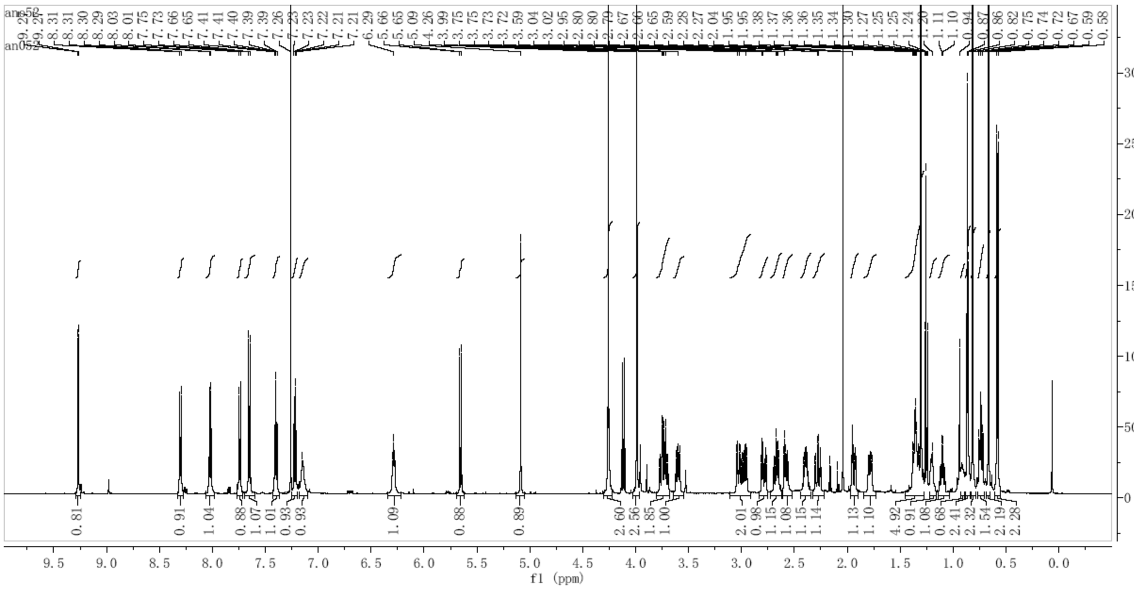

Figure S14. ^{13}C-NMR spectra of 7e.

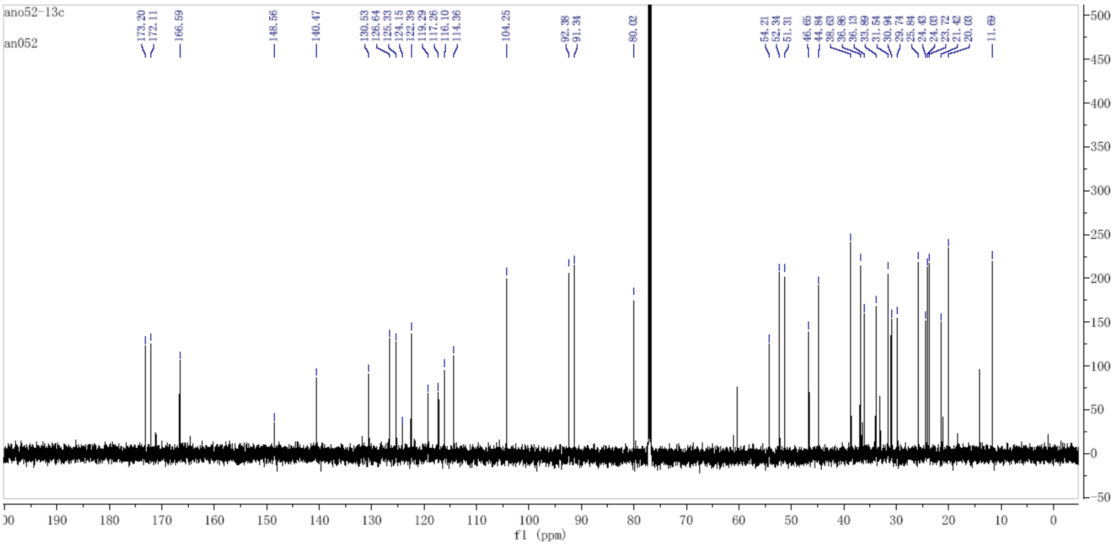

Figure S15. HRMS of 7e.

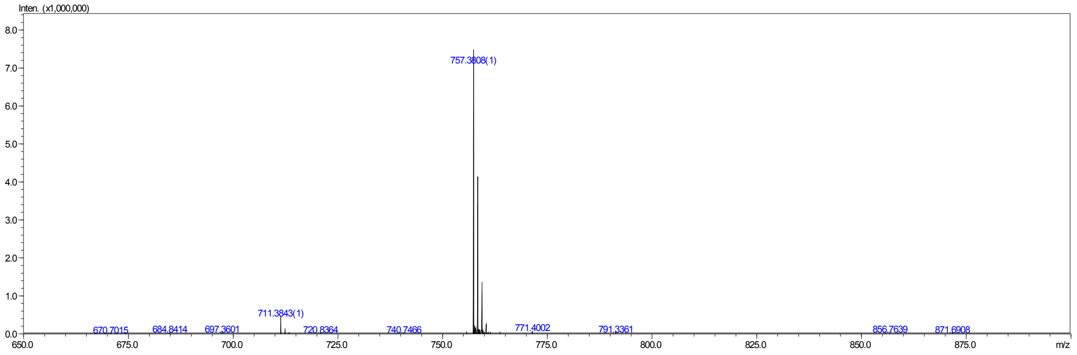

Figure S16. ^{1}H-NMR spectra of 7f.

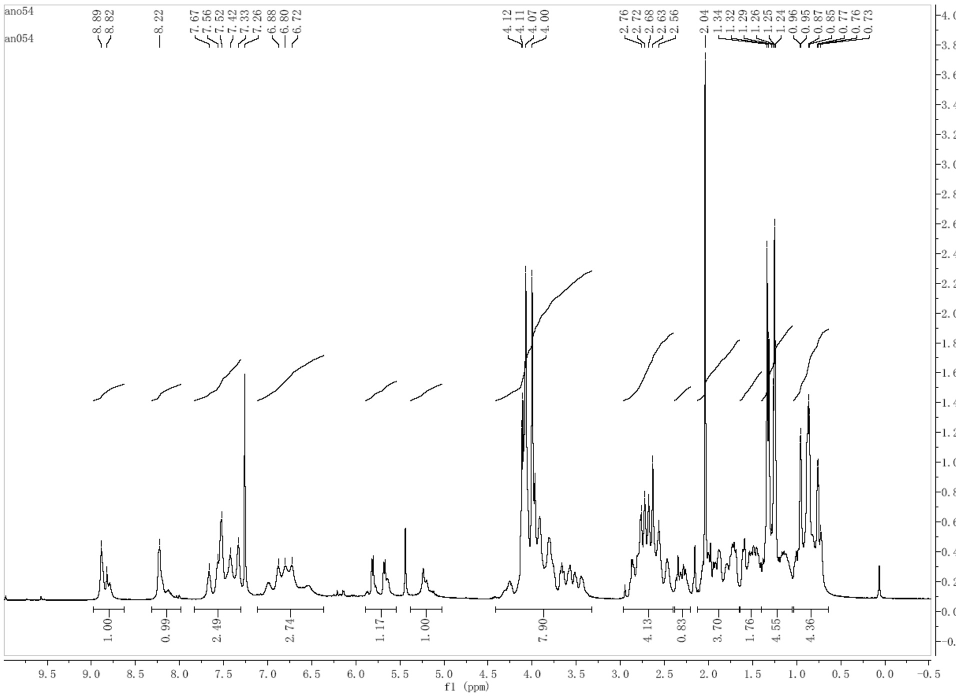

Figure S17. ^{13}C-NMR spectra of 7f.

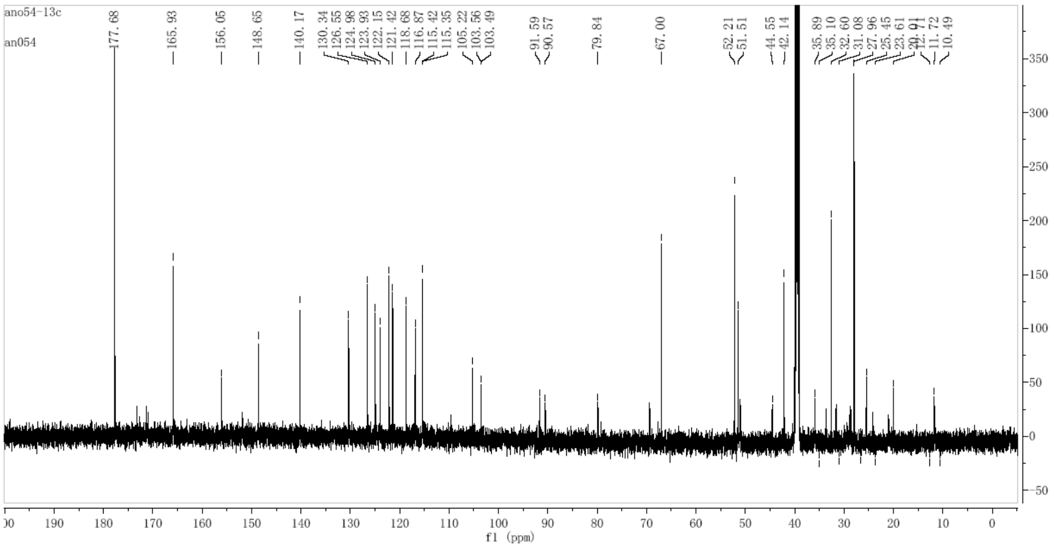

Figure S18. HRMS of 7f.

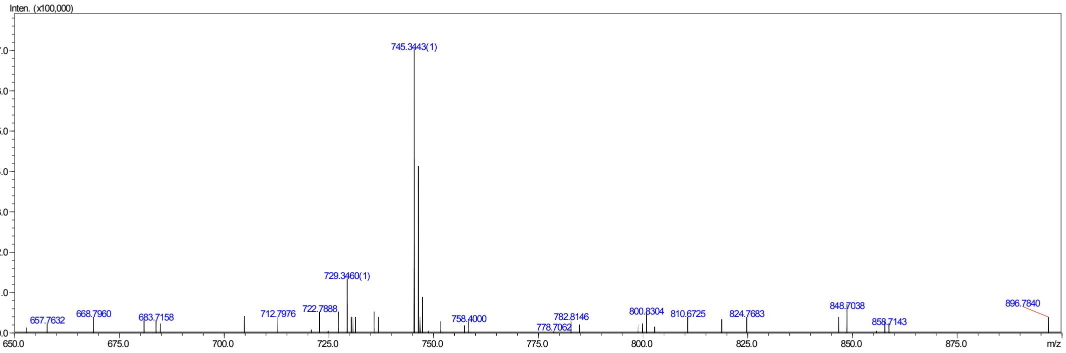

Figure S19. ^{1}H-NMR spectra of 8.

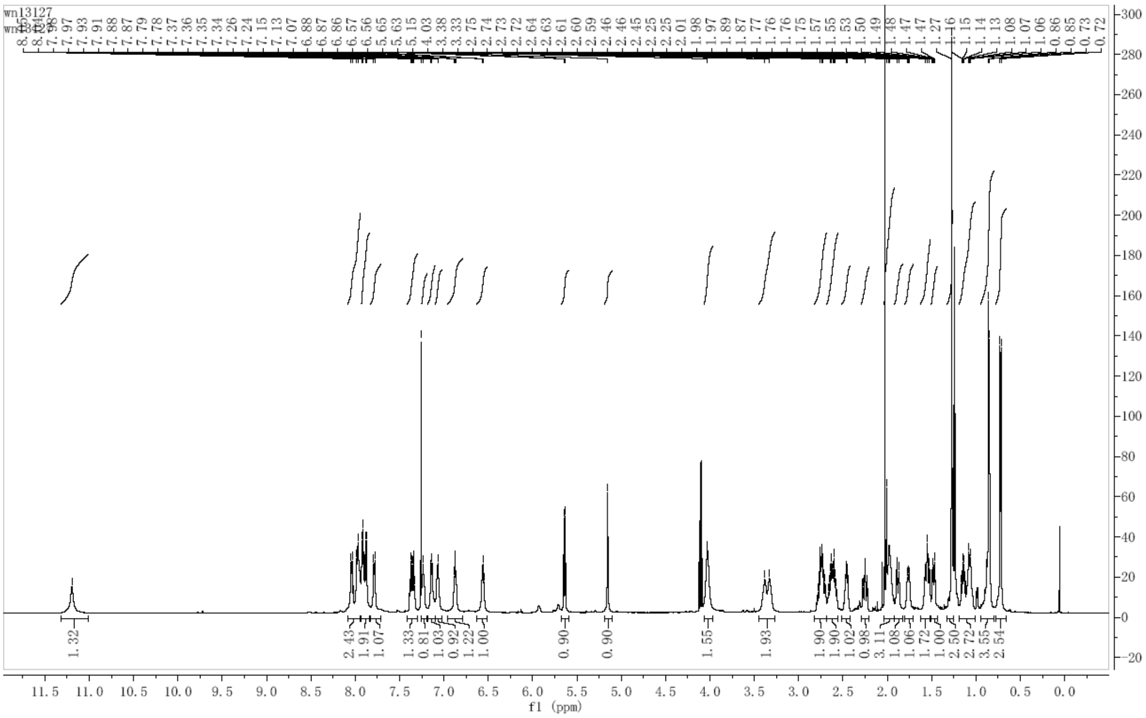

Figure S20. ^{13}C-NMR spectra of 8.

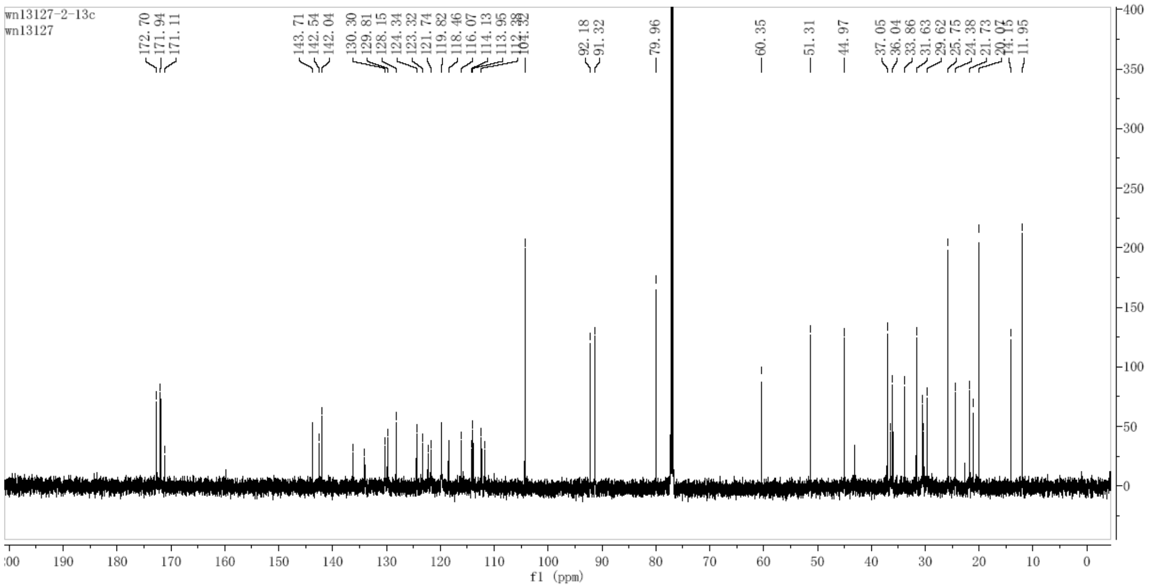

Figure S21. HRMS of 8.

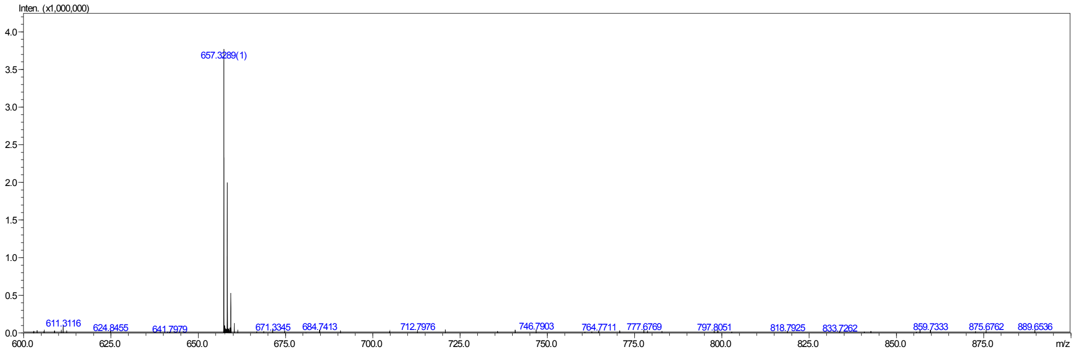

Figure S22. ^{1}H-NMR spectra of 9.

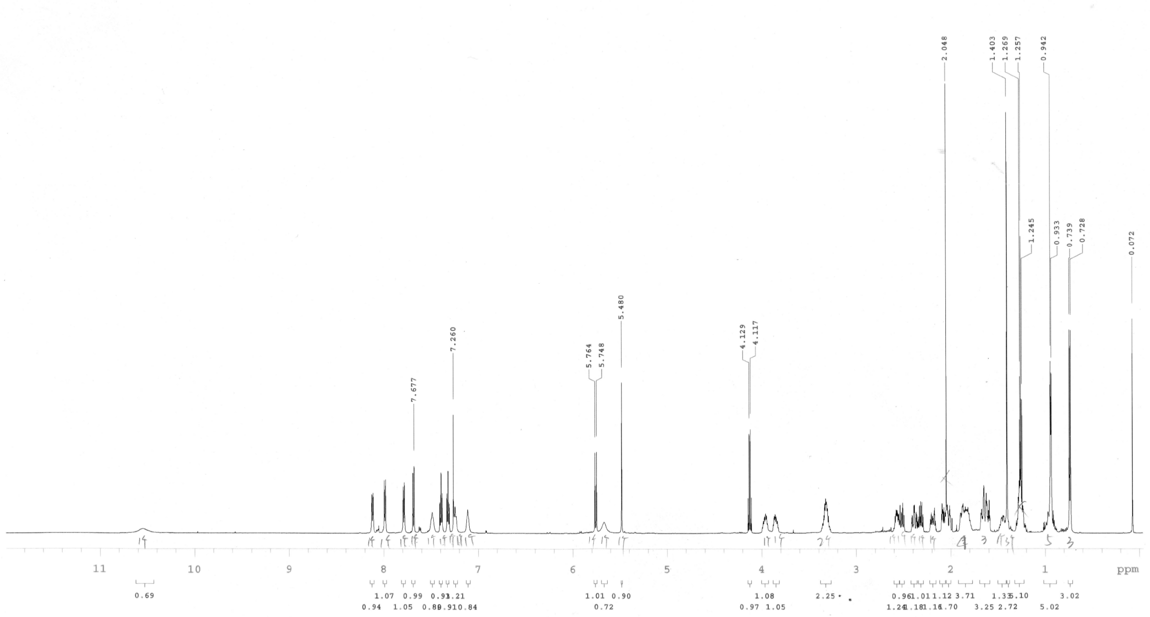

Figure S23. ^{13}C-NMR spectra of 9.

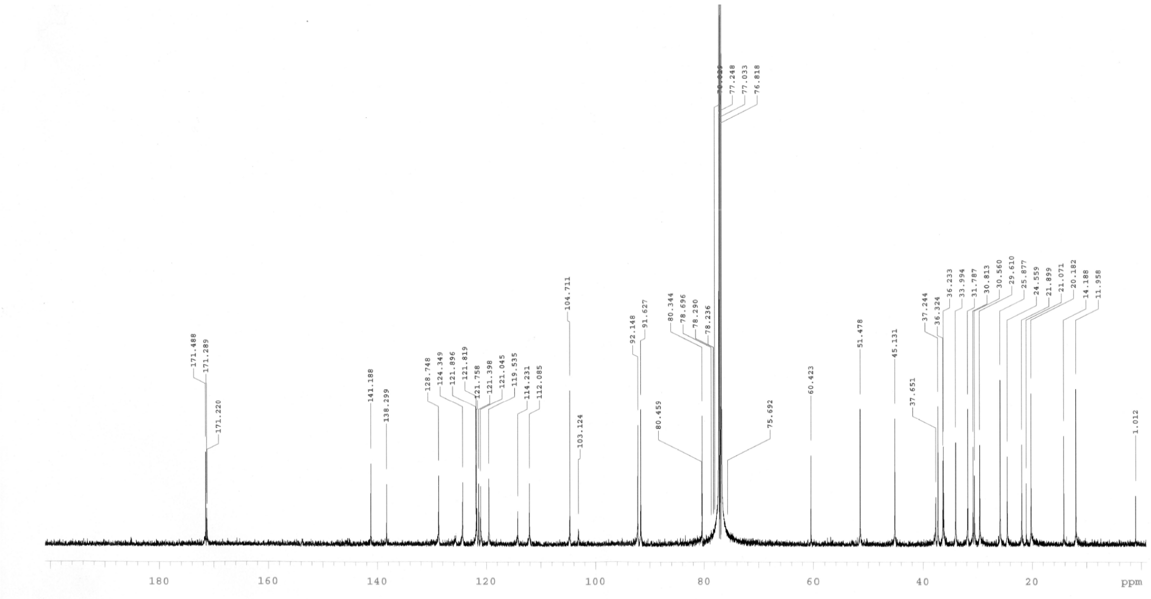

Figure S24. HRMS of 9.

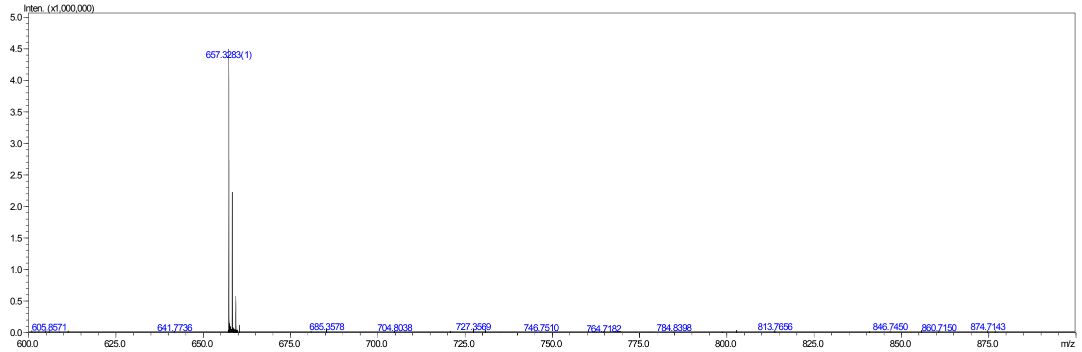

Supplement: Supplementary File 1 [file molecules-19-19021-s001.pdf]
